# Long-Term Potentiation in the CA1 Hippocampus Induced by NR2A Subunit-Containing NMDA Glutamate Receptors Is Mediated by Ras-GRF2/Erk Map Kinase Signaling

**DOI:** 10.1371/journal.pone.0011732

**Published:** 2010-07-22

**Authors:** Shan-xue Jin, Larry A. Feig

**Affiliations:** Department of Biochemistry, Tufts University School of Medicine, Boston, Massachusetts, United States of America; INSERM U901, France

## Abstract

**Background:**

NMDA-type glutamate receptors (NMDARs) are major contributors to long-term potentiation (LTP), a form of synaptic plasticity implicated in the process of learning and memory. These receptors consist of calcium-permeating NR1 and multiple regulatory NR2 subunits. A majority of studies show that both NR2A and NR2B-containing NMDARs can contribute to LTP, but their unique contributions to this form of synaptic plasticity remain poorly understood.

**Methodology/Principal Findings:**

In this study, we show that NR2A and NR2B-containing receptors promote LTP differently in the CA1 hippocampus of 1-month old mice, with the NR2A receptors functioning through Ras-GRF2 and its downstream effector, Erk Map kinase, and NR2B receptors functioning independently of these signaling molecules.

**Conclusions/Significance:**

This study demonstrates that NR2A-, but not NR2B, containing NMDA receptors induce LTP in pyramidal neurons of the CA1 hippocamus from 1 month old mice through Ras-GRF2 and Erk. This difference add new significance to the observation that the relative levels of these NMDAR subtypes is regulated in neurons, such that NR2A-containing receptors become more prominent late in postnatal development, after sensory experience and synaptic activity.

## Introduction

A large body of evidence has implicated NMDA-type glutamate receptors (NMDARs) in the process of learning and memory, at least in part, due to their contribution to long-term changes in synaptic strength in the forms of long-term potentiation (LTP) and long-term depression (LTD) [1]. NMDARs consist of dimers of calcium permeable NR1 subunits bound to either homo-dimers or hetero-dimers of regulatory NR2 subunits, NR2A-D. The rational for the existence of distinct subsets of receptors containing different NR2 subunits has been the subject of much controversy and remains poorly understood. In particular, some studies claimed that NR2A-containing receptors are specific for LTP induction, while NR2B receptors are specific for LTD induction [2], [3]. However, other studies have contradicted these findings [4], [5], [6] and support instead the idea that the magnitude of calcium influx through either channel is the critical determinant in generating a specific form of synaptic plasticity, with LTP requiring more calcium than LTD [4], [6].

One undisputed distinction between NMDAR subtypes is their calcium channel gating properties. NR2B containing receptors are slower to deactivate and therefore may carry more calcium per unit current than NR2A receptors (for review see [7]). This has led to the hypothesis that NR2B receptors induce LTP more easily than NR2A receptors, and thus the NR2A/NR2B ratio may control LTD/LTP thresholds. This model leaves open the possibility that the NR2A/NR2B ratio in synapses also influences qualitative differences in how LTP is induced via subunit-specific coupling to distinct intracellular signal transduction pathways [8] that remain poorly understood.

Previous studies have shown that the Ras-GRF family of calmodulin-binding exchange factors, Ras-GRF1 (GRF1) and Ras-GRF2 (GRF2), are calcium sensors that distinguish between LTP and LTD-inducing signals in the CA1 hippocampus, beginning at about 1-month of age in mice [9]. This idea is based on the observations that GRF2 knockout mice display defective LTP, but not LTD and GRF1 knockout mice display defective LTD but not LTP, when field excitatory postsynaptic potentials (fEPSPs) were used to measure synaptic plasticity. In addition, this study showed that NMDAR signaling to Erk MAP kinase, a known promoter of LTP is mediated by GRF2, while NMDAR signaling to p38 MAP kinase, a known promoter of LTD, is mediated by GRF1 in hippocampal brain slices. Finally, chemical inhibitors with selectivity for NR2A or NR2B receptors blocked NMDA activation of Erk and p38, respectively. These findings suggested that GRF2 mediates NR2A receptor function, while GRF1 mediates NR2B receptor function. However these studies did not directly demonstrate which NMDAR subtypes contribute to LTP induced by GRF2, nor did they address the question of whether NR2B receptors can also induce LTP. Here, using single cell recording from CA1 pyramidal neurons, we show directly that both NR2A and NR2B receptors can induce LTP at this synapse. However, NR2A containing receptors induce LTP through Ras-GRF2 and Erk Map kinase and NR2B containing receptors induce LTP through neither.

## Results

### Normal LTP is induced after pairing low frequency synaptic stimulation with postsynaptic depolarization (LFS pairing) in CA1 pyramidal neurons of 1-month old Ras-GRF2 knockout mice

**Figure 1:**
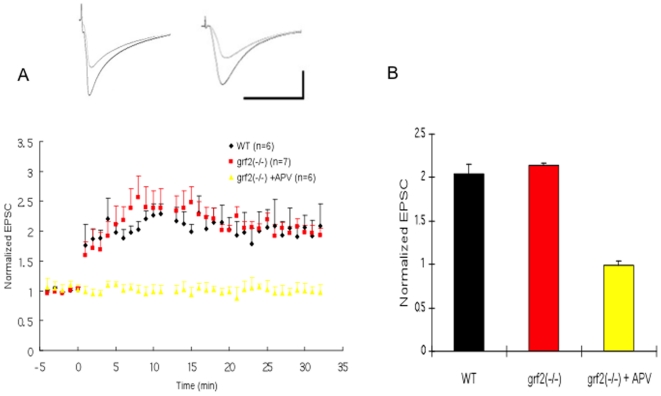
LTP induced by LFS paring protocol is normal in Ras-GRF2 knock-out mice. A. Low frequency stimulation (LFS paring) was used to induce LTP in P26-35 wild-type mice (black filled circles; n = 6) and Ras-GRF2 knock-out mice [grf2(−/−), red filled squares; n = 7] and Ras-GRF2 knock-out mice (yellow filled triangles; n = 6) in presence of 100µM APV. Insets, representative EPSC recorded before and after LFS pairing in wild-type (left) and grf2(−/−) (right) slices. Calibration: horizontal, 50 ms; vertical, 50 pA. B Histogram showing no effect of Ras-GRF2 on LTP (p>0.05 vs. WT) but complete inhibition using the NMDAR inhibitor APV.

We showed previously that 1-month old Ras-GRF2 knockout mice display defective LTP when their Schafffer collateral fibers were stimulated with theta-burst stimulation (TBS) and synaptic activity was probed at CA3/CA1 synapses using extracellular field recordings [9]. Surprisingly, here we found normal LTP in hippocampal brain slices of Ras-GRF2 knockout mice when single-cell recordings of CA1 pyramidal neurons in these brain slices were obtained after low frequency synaptic stimulation paired with postsynaptic depolarization (LFS pairing) (Fig. 1).

A recent study by Berberich [10] et al suggested a possible explanation for this apparent contradiction. They found that the requirement for individual NMDA receptor subtypes for LTP induction in 28-day old mouse CA1 pyramidal neurons depends how LTP is induced. In particular, high frequency stimulation (HFS) of afferent fibers followed by extra-cellular field recording required both NR2A and NR2B subclasses of NMDA receptors to be functional. They came to this conclusion using inhibitors with preference for NR2A (NVP-AAM077 (NVP)) or NR2B (Ro25-6981 (Ro) containing receptors. The latter show high degree of specificity, whereas the former must be used a low concentrations to exploit there modest ∼10-fold specificity [11]. Thus, treatment of brain slices with low doses of either the NR2B inhibitor, or the NR2A inhibitor partially blocked LTP induction, while treatment with both inhibitors completely blocked LTP.

In contrast, they found that an LFS pairing-induced LTP measured in single cells required only that either NR2A or NR2B type receptors be functional, most likely because this stimulation protocol is more intense than HFS stimulation of afferent fibers, and as such leads to sufficient calcium influx to induce LTP through either receptors subtype. Thus, treatment with low doses of either NR2A or NR2B inhibitors alone had no effect on LTP. Only when they were added together was complete inhibition of LTP observed.

Thus, our previous observation that GRF2 knockout mice display defective LTP using theta-burst/field recording, but not here using the LFS/single-cell recording protocol, suggested that Ras-GRF2 induces LTP by mediating the action of one, but not both subtypes of NMDA receptors in CA1 pyramidal neurons.

### Defective LFS pairing-induced LTP is observed in CA1 pyramidal neurons of GRF2 knockout mice when NR2B, but not NR2A, receptors are blocked

**Figure 2:**
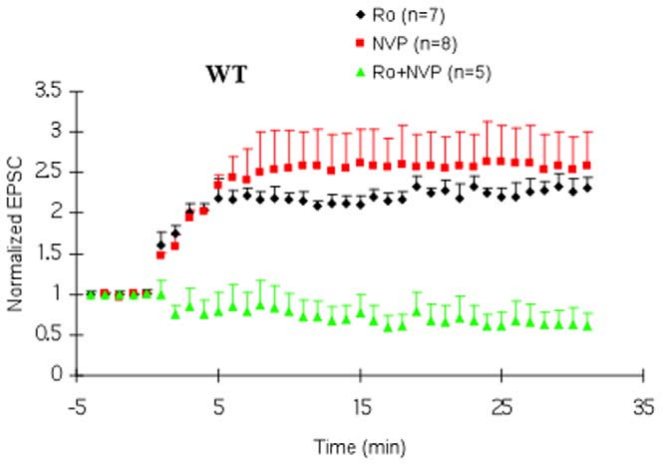
Effects of NR2B and NR2A-selective antagonists on LFS paring-induced LTP in WT mice. Hippocampal brain slices from WT mice were preincubated for 30 minutes with either 0.5 µM Ro25-6981(Ro) (black filled circles), 50 ηM NVP -AAM077 (NVP) (red filled squares) or both drugs (green filled triangles). LFS paring-induced LTP was then measured. Neither of the inhibitors had a significant effect on basal synaptic transmission (Suppl. Fig.S1).

To determine which NMDA subtypes function through Ras-GRF2, we first attempted to reproduce the major findings of Berberich et al in our system. Fig. 2 shows, as expected, that pre-treatment of hippocampus slices from wild type 28 day-old mice with either 0.5 µM R025-6981 or 50 µM NVP-AAM077 alone had no effect on LFS induced LTP. However treatment of brain slices with both inhibitors completely blocked LTP. Neither of the inhibitors blocked baseline response (Fig. S1)

**Figure 3:**
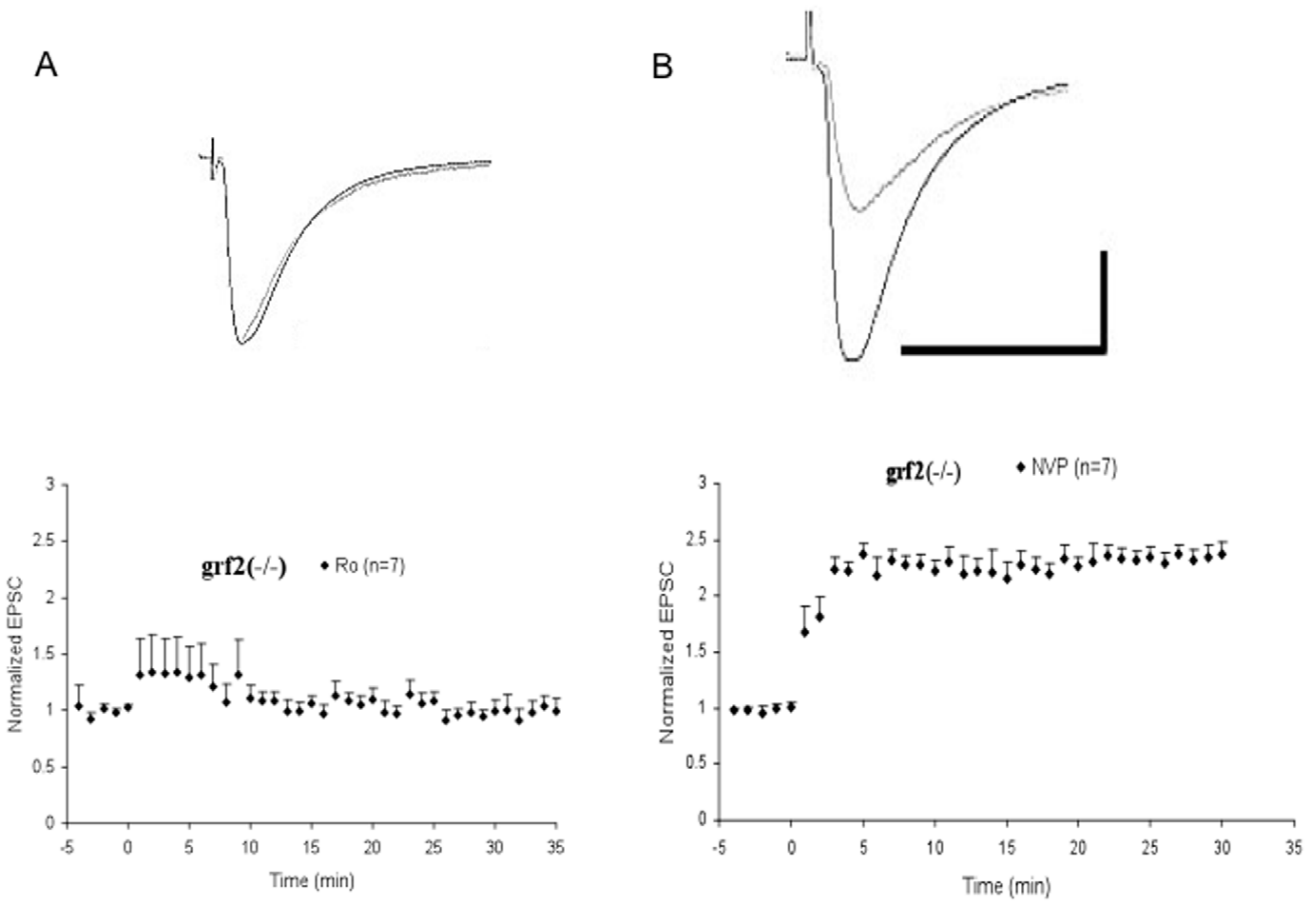
Effects of NR2B and NR2A-selective antagonists on LFS paring-induced LTP in Ras-GRF2 knock-out mice. **A**. Hippocampal brain slices from Ras-GRF2 knockout mice were perfused throughout the experiments with 0.5 µM of the NR2B selective inhibitor Ro. LFS paring-induced LTP was then measured. **B**. Hippocampal brain slices from Ras-GRF2 knockout mice were perfused throughout the experiments with 50 ηM of the NR2A selective inhibitor NVP. LFS paring-induced LTP was then measured. Inset: Averaged EPSCs before and after LFS paring in the presence of Ro (left) and NVP (right). Calibration: horizontal, 50 ms; vertical, 50 pA.

Then, the LFS paradigm was used on hippocampal brain slices from Ras-GRF2 knockout mice in the presence of either the NR2B inhibitor, which leaves NR2A receptors to induce full LTP, or the NR2A inhibitor, which leaves only NR2B receptors to induce full LTP in this system Fig. 3 shows that pretreatment of these mutant brain slices with the NR2B inhibitor severely suppressed LTP, demonstrating that Ras-GRF2 knockout mice have defective NR2A receptor signaling. In contrast, pretreatment with the NR2A inhibitor, NVP, had no effect, which implies that Ras-GRF2 knockout mice retain functional NR2B induced LTP. These findings strongly support the idea that Ras-GRF2 mediates LTP induced by NR2A, but not by NR2B or NR2A/2B NMDA receptors.

**Figure 4:**
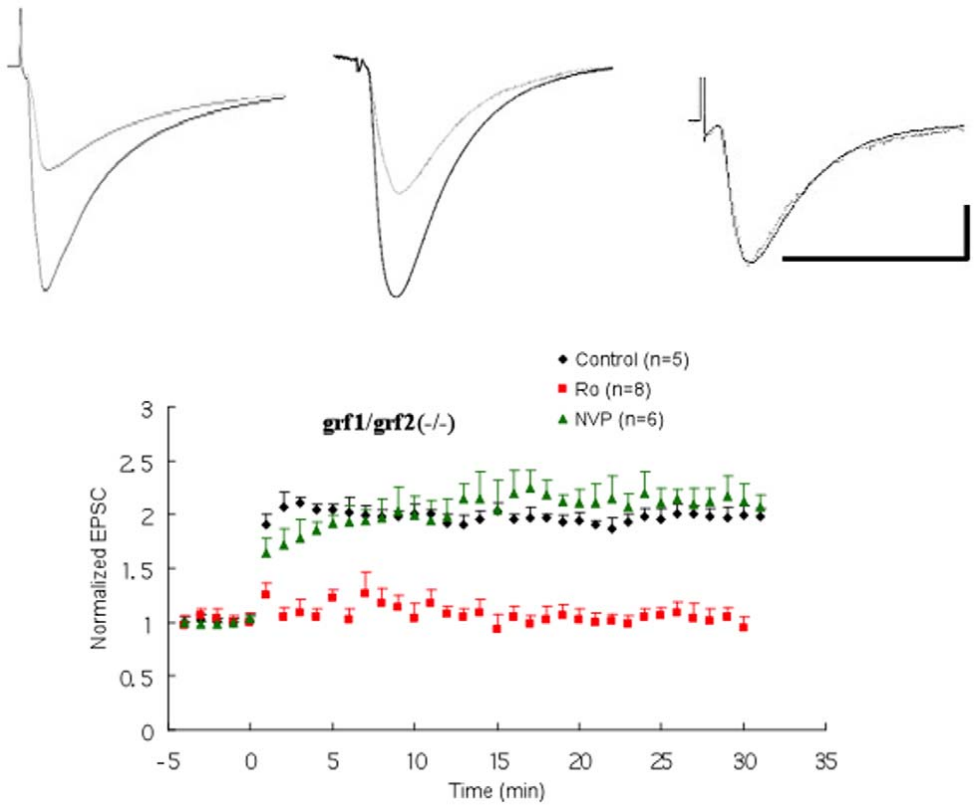
LFS paring-induced LTP in double Ras-GRF1/2 knock-out mice. LFS paring-induced LTP was measured in hippocampal brain slices from double Ras-GRF1/Ras-GRF2 knockout mice in normal (black filled circles; n = 5) and in the presence of 50 ηM NVP (green filled triangles; n = 6) or 0.5 µM Ro (red filled squares; n = 8) ACSF. Inset: Averaged EPSCs before and after LFS paring in control (left) and in the presence of NVP (middle) or Ro (right) ACSF. Calibration: horizontal, 50 ms; vertical, 50 pA.

We showed previously that GRF1, which is known associate with NR2B receptors, mediates LTD but not TBS-induced LTP as measured by field recordings in the CA1 hippocampus of 1-month old mice [9]. Fig. 4 shows that when LTP was induced by the LFS-pairing protocol in single cells of brain slices from double GRF1/GRF2 knockout mice, where NR2A/GRF2/LTP signaling is blocked, normal LTP was still observed. In contrast, pre-incubation with the NR2B inhibitor led to the loss of LTP induction, while pre-incubation with the NR2A inhibitor had no effect. These results confirms that although GRF1 associates with NR2B receptors, NR2B-induced LTP functions through neither Ras-GRF1 nor Ras-GRF2.

### NR2A but not NR2B receptors function through Erk Map kinase signaling

**Figure 5:**
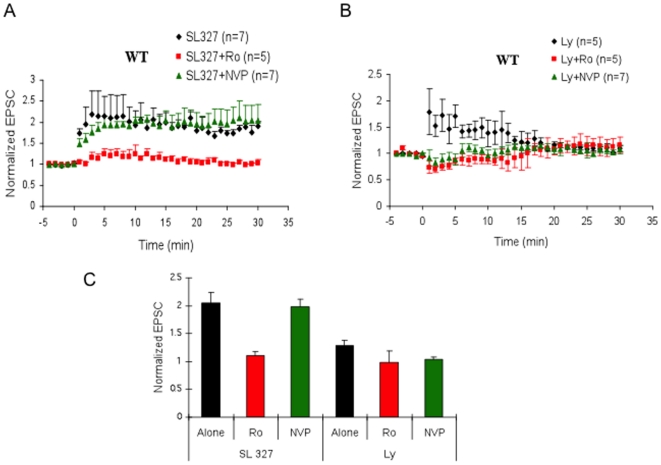
The effects of MEK and PI3-kinase inhibitors on LFS induced LTP in WT mice. **A** LTP was measured in cells in which SL327 (100 µM) was present in recording electrode solution (black filled circles; 0.2% DMSO; n = 7). Tissues were bathed in either 0.5 µM Ro (red filled squares; n = 5) or 50 ηM NVP (green filled triangles; n = 7). **B** LTP was measured in cells in which LY294002 (Ly) (100 µM) was included in the recording electrode solution (black filled circles; n = 5). These tissues were also bathed in either 0.5µM Ro (red filled squares; n = 5) or 50 ηM NVP (green filled triangles; n = 7). C. Summary graph showing the effects of these drugs on the LTP.

We then used a similar strategy to investigate whether a known downstream effector of Ras-GRF2, Erk Map kinase, also plays a sub-type specific role in NMDA receptor signaling. In particular, we tested the effect of including in the recording electrode, SL327, an inhibitor of Mek kinase, the upstream activator of Erk Map kinase. Fig 5A shows that SL327 treated neurons display normal LTP (Fig 5A; black diamond and 5C), implying that either Erk activation is not important for LFS-induced LTP, or that it selectively contributes to either NR2A or NR2B receptor signaling but not both. To distinguish between these possibilities, we included in the bath solution either an NR2A or NR2B inhibitor. We found that SL327 suppressed LTP induction when NR2B, but not NR2A receptors were blocked. Thus, like Ras-GRF2, Erk activity is needed for NR2A, but not NR2B, -induced LTP in CA1 pyramidal neurons of the hippocampus from 1-month old mice.

Not all signaling molecules known to be important for LTP show this receptor selectivity because the PI3-kinase inhibitor, LY294002 (Ly) suppressed LFS pairing-induced LTP even without treatment with either NR2A or NR2B inhibitors, indicating that PI-3 kinase contributes to both NR2A and NR2B-mediated LTP in these neurons (see Fig. 5B and 5C).

## Discussion

**Figure 6:**
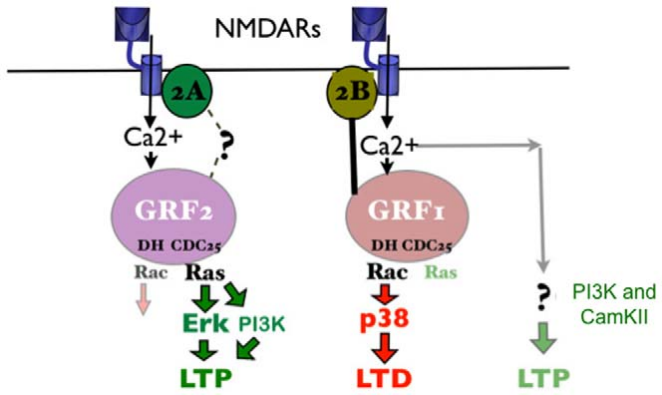
Model of Ras-GRF2 dependent and Ras-GRF independent LTP in hippocampal CA1 pyramidal neurons. Both NR2A and NR2B-containing NMDARs can produce LTP. However, GRF proteins only make a major contribution to LTP through NR2A-containing receptor signaling through GRF2. Although GRF1 binds to NR2B receptors and contributes to LTD, it does not contribute significantly to NR2B induced LTP. Moreover, like GRF2, Erk mediates only NR2A induced LTP, while PI3K mediates both NR2A and NR2B induced LTP.

This study demonstrates that NMDA receptors can promote LTP through at least two distinct signaling cascades in CA1 pyramidal neurons of the hippocampus of 1 month-old mice. One pathway involves NR2A-containing glutamate receptors, the guanine nucleotide exchange factor GRF2, and its downstream effector Erk Map kinase, while the other involves NR2B-containing receptors and neither GRF1, GRF2 nor Erk Map kinase (see Fig. 6). These conclusions are based on whole-cell recordings from GRF2 knockout hippocampal brain slices using low frequency stimulation coupled with depolarization of the post-synaptic cell (LFS pairing). This stimulation paradigm generates enough calcium influx through either NR2A or NR2B receptors to invoke a full LTP in the CA1 hippocampus [10]. As such, inhibition of any protein that mediates the action of only one of these receptors, like GRF2, will have no effect on this type of LTP induction paradigm. LTP will be inhibited only when another pathway is blocked either genetically or with a chemical inhibitor. In this case, we found that the NR2B-specific inhibitor R025-6981 did not suppress LTP in wild type mice, but did suppress LTP in GRF2 knockout CA1 neurons, indicating that GRF2 mediates NR2A receptor function. Since RO25-6981 does not efficiently block receptors containing heteromers of NR2A/NR2B [11] subunits, yet LTP was blocked, this class of NMDA receptor does not make a significant contribution to LTP at this synapse. Consistent with this conclusion, NVP-AAM077, an inhibitor that displays some preference for NR2A receptors, did not block LTP in GRF2 knockout neurons. Moreover, the same results were obtained when these inhibitors were used to study LTP in NR2A knockout mice [10]. In contrast, it has been shown that theta-burst stimulation of LTP measured by extracellular field recordings requires both NR2A and NR2B receptors to be functional [10], which explains why we found previously [9] that GRF2 knockout mice display decreased LTP induced by this stimulation paradigm, even in the absence of any inhibitor.

One possible mechanism for the specific coupling of GRF2 to NR2A receptors could be physical association of the two proteins, which would localize the calmodulin-binding motif of GRF2 close to a calcium source entering neurons through the NR1 subunit of NR2A-containing receptors. However, we could not document stable association of GRF2 with NR2A receptors after immunoprecipitation (data not shown) suggesting that GRF2 is physically near NR2A receptors in the membrane but not tightly bound to it. Interestingly, tight association of a calcium responsive signaling protein to a specific calcium permeating receptor cannot account for all of the specificity observed because GRF1 binds directly and tightly to NR2B receptors, but we show here that it does not mediate NR2B-induced LTP in CA1 pyramidal neurons in 1-month old mice. Instead, we showed previously that it mediates LTD, most likely because of its ability to activated p38 Map kinase, not Erk Map kinase [9].

One of the best-characterized downstream effector pathways regulated by GRF2 is that made up of Ras, Raf, and Erk Map kinase [9], [12]. Like GRF2-inhibited hippocampal brain slices obtained from knockout mice, Erk inhibited slices showed no defect in LFS pairing-induced LTP unless samples were preincubated with NR2B, but not NR2A, inhibitors. Thus, like GRF2, Erk signaling is needed for NR2A, but not NR2B, receptor-mediated LTP. These results are consistent with a previous study connecting Erk signaling with NR2A receptors [13]. Erk contribution to late LTP is known to function through its role in promoting CREB mediated gene expression [14] [15], [16], [17]. Interestingly, another downstream target of Erk is the transcription factor fos, which has also been implicated in NR2A, but not NR2B,-mediated LTP [18].

GRF2 has the potential to influence LTP by regulating many other signaling pathways besides Erk Map kinase, since Ras can activate effectors other than Raf kinase, such as PI3 kinase and Ral-GDS. In addition, GRF2 has a Rac activating DH domain that has the potential to regulate many signaling pathways that regulate other Map kinases as well as the cytoskeleton. However, not all signaling molecules contribute to LTP in a receptor-subtype specific manner, since a PI3-kinase inhibitor blocked LFS pairing induced LTP without the need to block either receptor subtype, indicating it contributes to both NR2A and NR2B mediated LTP.

There is growing interest in the functional differences between LTP induced by NR2A and NR2B-containing NMDARs as more evidence accumulates showing that the ratio of these subclasses of receptors in NMDARs is regulated by metaplasticity, where a synapse's previous history of activity determines its current plasticity. For example, the ratio of receptor subtypes is known to change during postnatal development [19], [20]. NR2B receptors predominate in the neonatal period and early development, while NR2A subunit expression increases with age and becomes the dominant subunit after puberty. This pattern of NR2A expression coincides with the developmentally-regulated expression pattern of GRF2, which only participates in LTP regulation after ∼3–4 weeks of age in the mouse [9]. Thus, both NR2A and GRF2 become dominant participants in LTP just when the hippocampus begins to make a major contribution to learning and memory [21], [22]. These correlations suggest that GRF-independent, NR2B-mediated LTP may be more important for LTP associated with the development of the hippocampus, while GRF2 and Erk-dependent NR2A-induced LTP is primarily involved in memory contributions of the mature hippocampus.

Another example of metaplastic regulation of NMDAR subunit composition occurs in the visual cortex where sensory experience increases the NR2A/NR2B ratio at synapses (for reviews see [7], [23]). Similarly, in the CA1 hippocampus of young but not adult animals, the NR2A composition can be increased dramatically by NMDAR activation [23]. In particular, induction of LTP leads to a rapid replacement of NR2B receptors with NR2A receptors. Overall these studies highlight the need to reveal the unique contributions that NR2A subunits make to NMDAR-mediated LTP. Future experiments that lead to a full understanding of how GRF2 leads to LTP by these receptors will make a significant advance toward this goal.

## Methods

### Mice

Ras-grf2(−/−) and double ras-grf1/ras-grf2(−/−) mice were described previously (Tian et al. 2004, Li et al. 2006). Mice were maintained on a mixed C57BL/6Jx129 genetic background. Wild-type (WT) mice of the same background were also used. All maintenance and procedures concerning mice were in accordance with the animal welfare guidelines of Tufts University.

### Extracellular field experiments

Mutant and control mice (P26-35) were anesthetized with halothane and decapitated. The transverse acute hippocampal slices (350 µm) were cut in ice-cold oxygenated sucrose-enhanced artificial cerebrospinal fluid (ACSF) containing 206 mM sucrose, 2 mM KCl, 2 mM MgSO_4_, 1.25 mM NaH_2_PO_4_, 1 mM CaCl_2_, 1 mM MgCl_2_, 26 mM NaHCO_3_, 10 mM D-glucose, pH 7.4. After dissection, slices were incubated in ACSF that contained the following (in mM): 124 NaCl, 2 KCl, 2 MgSO_4_, 1.25 NaH_2_PO_4_, 2 CaCl_2_, 26 NaHCO_3_, 10 D-glucose, pH 7.4, in which they were allowed to recover for at least 90 min before recording. Recording were performed in the same solution, in a submerged chamber. To record field EPSPs (fEPSPs) in the CA1 region of the hippocampus, standard procedures were used. Schaffer collaterals were stimulated with an unipolar stimulating electrode (World Precision Instruments) placed in the lateral CA1 subfield. A borosilicate glass recording electrode filled with ACSF was positioned in stratum radiatum of CA1, 250–350 µm from the stimulating electrode. Data were presented as mean ± SEM, and statistical significance was evaluated using two-way ANOVA, followed by Student-Newman-Keuls post-hoc test.

### Whole-cell experiments

Mice were killed by halothane inhalation. The brain was removed quickly and submerged in ice-cold artificial CSF (ACSF) containing the following (in mM): 125 NaCl, 2.5 KCl, 1 MgCl_2_, 2 CaCl_2_, 25 NaHCO_3_, 1.25 NaH_2_PO_4_, and 25 D-glucose saturated with 95% O_2_ and 5% CO_2_ (pH7.4). Transverse slices (350 µm thickness) were cut in ice-cold ACSF with a vibratome (1000-plus sectioning system) and incubated in ACSF for 30 min at 35°C before recording.

CA1 pyramidal cells were visualized using infrared differential interference contrast microscopy (Nikon Eclipse E600FN, Tokyo, Japan) in combination with a newvicon tube camera (Dage-MTI, Michigan City, IN). Recording pipettes (3–6 MΩ) was filled with (in mM): 120 Cs-gluconate, 10 CsCl, 8 NaCl, 10 HEPES, 0.2 EGTA, 2 MgATP, 0.3 Na_3_GTP, 10 Phosphocreatine (pH 7.3). Series resistances (10 to 30 MΩ) and input resistances (100 to 300 MΩ) were monitored throughout the experiment using negative voltage steps (−5 mV, 20ms). The membrane potential was held at −70 mV. Experiments were performed at room temperature (21–24°C). The recording chamber was perfused at speed of 2 mL/min with (in mM): 124 NaCl, 2.5 KCl, 26 NaHCO_3_, 1.25 NaH_2_PO_4_, 4 CaCl_2_, 4 MgSO_4_, 10 D-glucose and 0.01 bicuculline methiodide (BMI) (pH 7.3). Excitatory postsynaptic currents (EPSCs) were evoked by extracellular stimulation of schaffer collaterals using a unipolar stimulating electrode (World Precision Instruments). Paring low frequency stimulations (120 pulses, 0.7 Hz) with postsynaptic depolarization to 0 mV for 3 min were used to induce LTP in patched cells. Liquid junction potentials were not corrected. Six consecutive EPSCs were averaged and normalized to the mean value recorded 5 min before conditioning stimulus. EPSC potentiation was assessed 30 min after conditioning stimulus. As for the drugs, D-APV (Sigma), Ro25-6981 (Sigma) and NVP-AAM077 (Novartis) were bath-applied after dilution into the ACSF, but LY294002 (Calbiochem) and SL327 (Sigma) were included in the recording electrode solution. Data were pooled across animals of the same treatment and genotype and presented as mean ± SEM. Two-tailed Student's t test or two-way ANOVA followed by post-hoc Student –Newman-Keuls test were used for evaluation of statistical significance.

## Supporting Information

Figure S1 Ro and NVP have no effects on the baseline response in slices from WT mice. A. 0.5 µM Ro was bath applied (n = 6). B. 50 nM NVP was bath applied (n = 5). Whole cell recordings were made for the indicated times.
(0.03 MB JPG)
