# Supplementary figures and images for: Long-Term Potentiation in the CA1 Hippocampus Induced by NR2A Subunit-Containing NMDA Glutamate Receptors Is Mediated by Ras-GRF2/Erk Map Kinase Signaling
Source: PLoS One. 2010 Jul 22;5(7):e11732. doi: 10.1371/journal.pone.0011732 (PMC2908693; doi:10.1371/journal.pone.0011732)

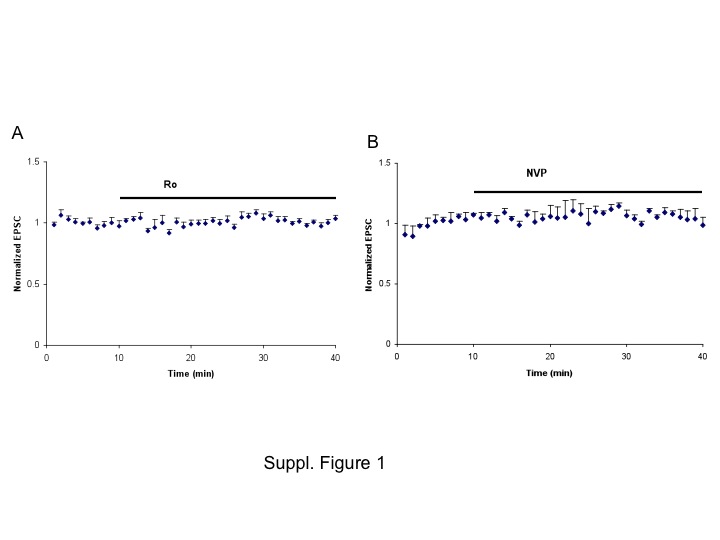

Supplement: Figure S1 — Ro and NVP have no effects on the baseline response in slices from WT mice. A. 0.5 µM Ro was bath applied (n = 6). B. 50 nM NVP was bath applied (n = 5). Whole cell recordings were made for the indicated times. (0.03 MB JPG) [file pone.0011732.s001.jpg]
